# Supplementary material for: Enhanced Stability and Compatibility of Montelukast and Levocetirizine in a Fixed-Dose Combination Monolayer Tablet
Source: Pharmaceutics. 2024 Jul 21;16(7):963. doi: 10.3390/pharmaceutics16070963 (PMC11279677; doi:10.3390/pharmaceutics16070963)
Supplement: Supplementary file 1 [file pharmaceutics-16-00963-s001.zip › pharmaceutics-3107250-supplementary.pdf]

Supplementary information

Enhanced Stability and Compatibility of Montelukast and Levocetirizine in a Fixed-Dose Combination Monolayer Tablet

Tae Han Yun[1], Moon Jung Kim[1], Jung Gyun Lee[1], Kyu Ho Bang [1,*] and Kyeong Soo Kim [1,*]

[1] Department of Pharmaceutical Engineering, Gyeongsang National University, 33 Dongjin-ro, Jinju 52725, Republic of Korea

*Corresponding author: Kyeong Soo Kim, Prof. Dr.
Tel: +82-55-772-3391
Fax: +82_55-772-3399
E-mail: soyoyu79@gnu.ac.kr

*Co-corresponding author: Kyu Ho Bang, Prof. Dr.
Tel: +82-772--3396
Fax: +82-772-3399
E-mail: khbang0095@gnu.ac.kr

(A)

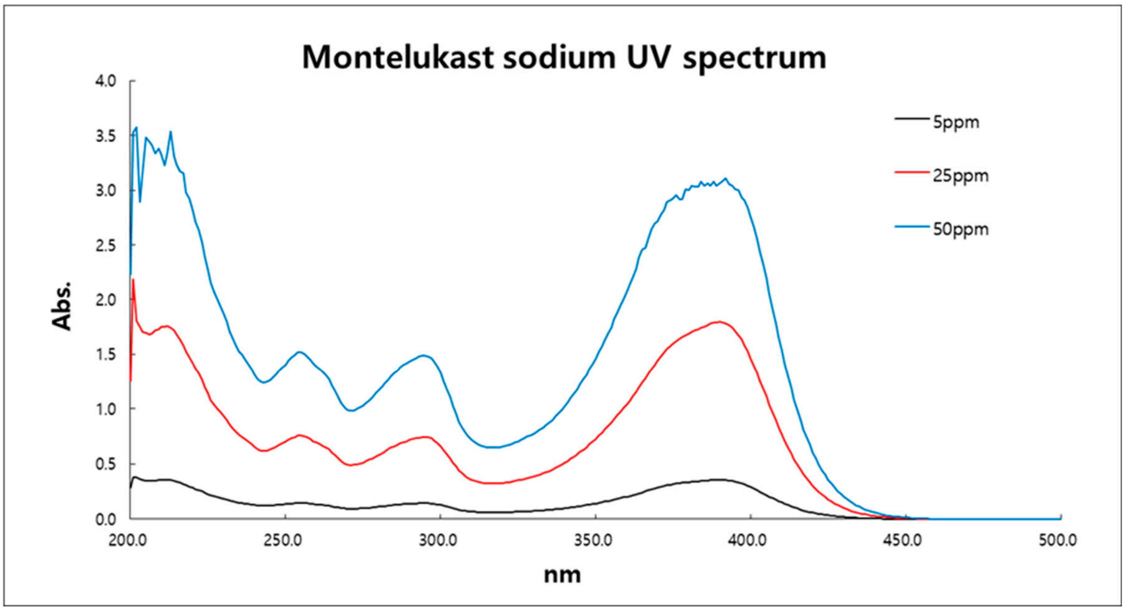

(B)

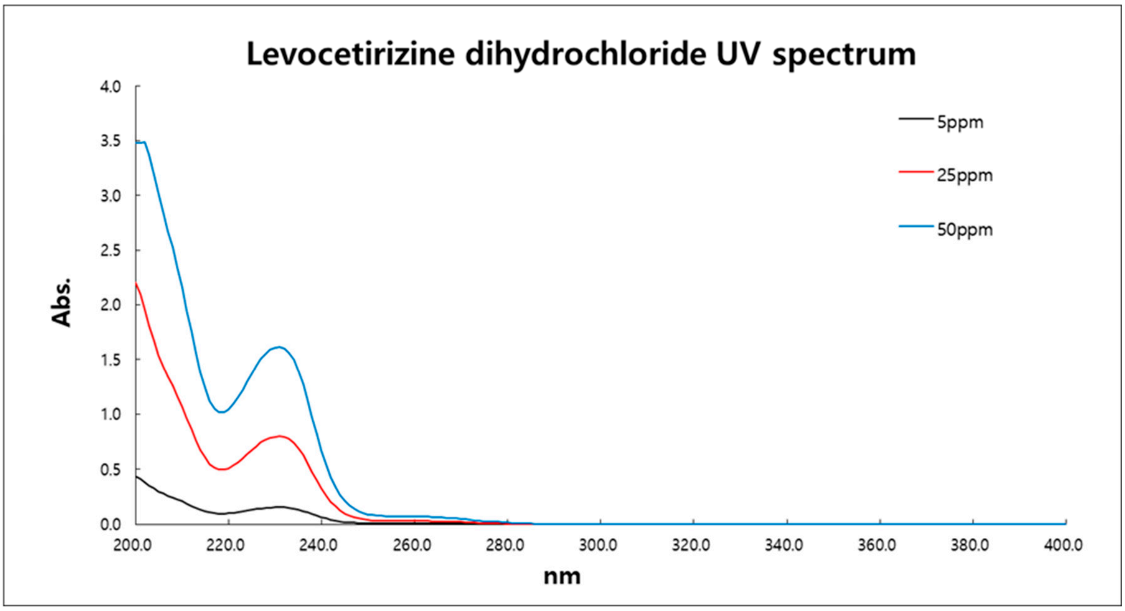

Figure. S1. UV Spectrum of (A) Montelukast and (B) Levocetirizine.

Table S1. Mechanical properties of initial F3 and F4 tablets.

Parameters	F3	F4
Mass variation (mg)	352.1 ± 2.2	355.3 ± 3.7
Hardness (KP)	8.5 ± 0.4	8.2 ± 0.8
Friability (%)	0.11 ± 0.04	0.10 ± 0.05
Disintegration time (s)	103 ± 16	110 ± 18
Content uniformity (MTL)	6.1	5.8
Content uniformity (LCZ)	7.4	8.2
